# Flour beetles prefer corners over walls and are slowed down with increasing habitat complexity

**DOI:** 10.1098/rsos.231667

**Published:** 2024-01-17

**Authors:** Inon Scharf, Amit Radai, Dar Goldshtein, Kimberley Hanna

**Affiliations:** School of Zoology, The George S. Wise Faculty of Life Sciences, Tel Aviv University, Tel Aviv 69978, Israel

**Keywords:** wall-following behaviour, habitat selection, movement ecology, obstacles, pest ecology, thigmotaxis

## Abstract

Movement affects all key behaviours in which animals engage, including dispersal and habitat use. The red flour beetle, known as a cosmopolitan pest of stored products, was the subject of our study. We examined whether the beetles preferred corners, walls or open areas, and how turns or obstacles in corridors delayed the beetles' arrival at a target cell. Beetles spent significantly more time in corners than expected by chance, while they spent considerably less time in open areas than expected. However, no significant difference was observed between areas with two or three surrounding walls. This could be attributed to the beetles' stronger attraction to corners than crevices or the insufficient proximity of the third wall to the other two. Movement through the corridor was delayed by turns or obstacles, expressed in arrival probabilities, arrival times, time in the corridor or movement speed. Obstacles on the corridor's perimeter had a stronger effect on the beetle movement than those in the corridor's centre owing to the beetles' tendency to follow walls. The research is important also for applied purposes, such as better understanding beetle movement, how to delay their arrival to new patches, and where to place traps.

## 1. Introduction

Movement is an integral part of many important behaviours. Several movement characteristics, like speed and directionality, affect foraging success, predation avoidance and successful dispersal [1–3]. When moving, animals trade off between the benefits and costs of movement. While movement allows animals to achieve specific goals such as finding food or mates, it is also energetically costly and poses various risks [4–6]. Animals rarely use their habitat uniformly and they prefer specific areas while avoiding others. For instance, animals exposed to predation risk often prefer sheltered areas over open ones [7,8]. Many vertebrate and invertebrate species favour moving along walls as opposed to open areas [9–12]. The preference for wall following is driven by various factors, and avoiding predation, alleviating anxiety and using the wall as a means of exploration or an exit are the most common explanations [13–16]. Movement along walls differs from movement in open areas in various aspects, such as increasing or decreasing speed upon reaching the wall (cf. [17,18]). Walls are not limited to artificial structures in human-made habitats, but they also include natural elements like rocks, wood trunks, fallen logs and other obstacles (e.g. [19,20]).

Habitat complexity increases with the abundance of surface irregularities, such as walls, rocks and wood trunks [21]. Habitat complexity greatly affects movement. For example, as complexity rises, movement becomes slower and less directional because animals cannot simply move forward [22–24]. Frequent turns not only slow movement but also elevate the energetic cost of movement [25,26]. Complexity, however, may contribute to predation avoidance and reduce competition with dominant species by increasing the number of available shelters and enabling the coexistence of more species [27–29]. The location and spatial distribution of obstacles in the habitat are important on top of their number. For example, with increasing complexity and especially when obstacles are clumped, obstacles tend to form corners (meeting points of two obstacles). There is less research on how animals relate to corners than walls, but a preference for corners has been detected in some systems, probably because animals feel safer there [30–33]. Animal behaviour in corners may differ from the behaviour along walls. Furthermore, if there is already a preference for movement in specific parts of the habitat, like movement along the habitat periphery, obstacles in such habitat parts or in front of the target, may have a more significant impact on animals than obstacles in less frequently used habitat parts [34,35].

Inter-sexual differences in movement behaviour and spatial orientation are common [36,37]. Referring to wall-following as an example, females tend to move along walls more than males [12,38,39]. Factors that enhance wall following, such as predation cues, have a weaker effect on females, possibly due to their already high preference for walls. By contrast, factors that moderate wall following, like certain drugs, may affect females more strongly than males [40,41].

In our study, we used the red flour beetle (*Tribolium castaneum* Herbst, 1797) as a model to examine the relative preference of beetles for walls and corners and their movement through corridors obstructed in different ways. Red flour beetles are important and common pests of stored products and mill storage [42,43], and they have served as a model organism in various biological fields [44,45]. The beetle disperses both by flying and walking [46–48]. Understanding the movement of this beetle is important for preventing dispersal among food patches and for optimizing trap deployment [49,50]. Previous studies demonstrated that red flour beetles strongly followed walls, even more than 80% of the time [51,52]. The beetle's preference depends also on arena size and structure, with walls being favoured to a greater extent in smaller and round arenas compared with larger or rectangular ones [35]. While some tests indicate that females tend to move more than males, this trend does not hold in all cases (cf. [35,51,53]). At least in this species, movement increases after eclosion and then decreases with age, decreases following starvation, but increases after mating [51,52,54,55].

Our study comprised three complementary experiments. In the first experiment, we examined beetle movement in arenas with squares containing varying numbers of walls: three, two, one and zero walls. We assessed whether movement differed among the different square types. In the second and third experiments, we investigated the beetle movement from an origin cell to a target cell through a corridor. The corridor was either straight or involved turns and was either free of obstacles or contained obstacles either in the centre or along the corridor's walls. We also explored potential inter-sexual differences in movement. Given the beetles' strong thigmotactic tendency (i.e. they tend to remain close to vertical surfaces like walls), we anticipated a preference for walls over open areas and corners in the arena. We expected that corridors necessitating turns would slow down the beetles and hinder their arrival at the target cell. Likewise, we expected obstacles along the corridor walls to have a more substantial impact on the beetle movement than obstacles located in the centre.

## 2. Methods

### 2.1. General methods

*Tribolium castaneum* beetles, collected in a mill storage in northern Israel in January 2020, have been maintained at Tel Aviv University in a climate cabinet set at 30°C (the optimal temperature [56]), with a 12 : 12 light : dark cycle, for at least 30 generations. A new generation was created by placing 500 random beetles in five boxes (diameter of 11 cm) filled with 120 g of a wheat flour–yeast mixture (ratio of 10 : 1). The beetles from the different boxes were mixed every three generations. The experiments were conducted between June and September 2023. Two weeks before the experiment, pupae were separated by sex, placed in a mixture of flour and yeast (ratio of 10 : 1), and allowed to emerge. Each experiment comprised 20 beetles, 10 of each sex. The first experiment comprised a single test, whereas the second and third ones comprised three treatments or three arena settings. Each beetle was tested under all treatments in random order. We glued white paper on the arena's surface to allow the beetles to move easily and to improve the contrast. For acclimatizion, each beetle was initially placed in the centre of the arena and covered with a tube lid (3 cm in diameter) for 30 s. Upon lid removal, we recorded the beetle's behaviour for 5 min using a Logitech Brio 4 K Ultra HD Webcam (30 frames per second (fps)). Between successive tests in the second and third experiments, the beetle was moved to a small plate with a bit of flour. Each dish was allowed to air out for approximately 30 min before reuse.

### 2.2. Experiment 1: do beetles stay longer in areas surrounded by walls?

**Figure 1.**
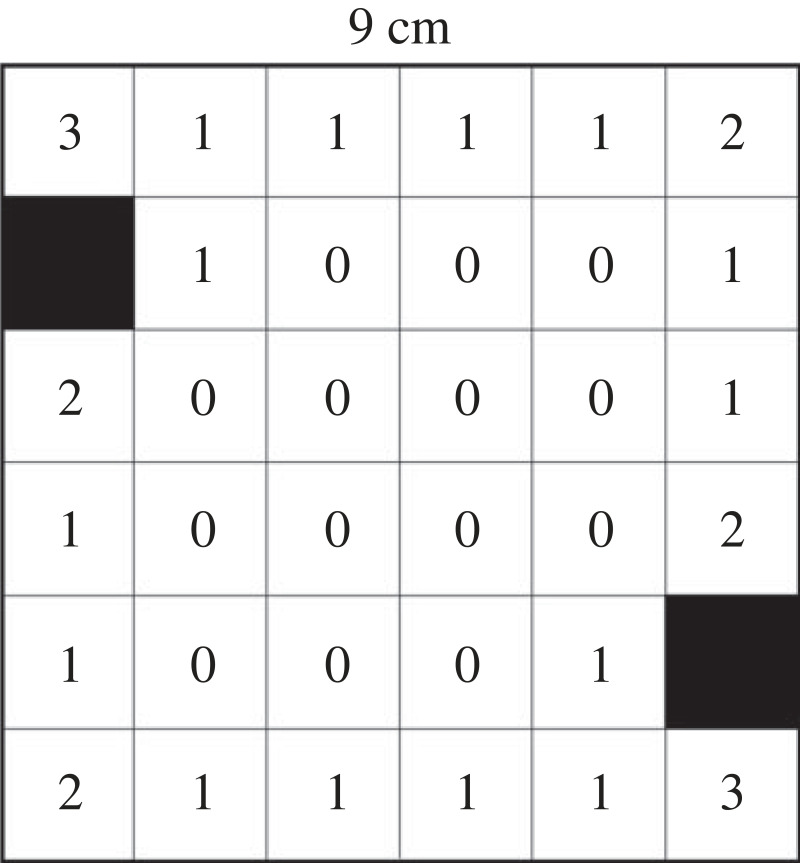
Scheme of the arena used in Experiment 1. Two blocks (1.5 × 1.5 cm; in black) were placed in the middle of a square arena (9 × 9 cm). The arena was virtually divided into 36 squares of which 34 were accessible to the beetles. Each square had either 0, 1, 2 or 3 surrounding walls (marked inside each square).

We employed a miniature version of the arena design described by Lamprea *et al*. [14], which consisted of a 9 × 9 cm arena with two 1.5 cm blocks placed near its upper left and lower right corners. The arena was divided into 36 (= 6 × 6) squares with differing numbers of walls (ranging from zero to three; figure 1), with 34 of these squares accessible to the beetles.

We encountered difficulties using automatic tracking software because the beetles occasionally disappeared behind the blocks. Therefore, we divided the video recordings into 300 frames (1 fps) and used ImageJ [57] to track the beetle and mark its location. We counted the number of frames in which each beetle was present in one of the four square types, categorized by the number of surrounding walls (0–3). The time the beetles spend in each square type can indicate the extent of their wall-following behaviour and/or preference for corners. Thus, we obtained four values per beetle. To investigate the influence of square type (or the number of surrounding walls) and sex on the time spent in each square type, we employed a repeated-measures ANOVA with square type as the within-subjects variable and sex as the between-subjects variable. Time was square-root transformed as it deviated from a normal distribution. There was no difference between the sexes so sex was removed and the test was redone. The number of squares per square type was not equal, with two squares having three walls, four with two walls, 14 with one wall and 14 with no walls (figure 1). We calculated the average time spent in each square type and compared it with the null expectation (equal time for each square) using a *χ*^2^-test. Next, we calculated the distance moved in each square and averaged it per beetle. We used a repeated-measures ANOVA to analyse distances, as described above, with distances square-root transformed. We were also interested in whether beetles were attracted to corners compared with walls. We summed the time beetles stayed in a radius of 0.5 cm from one of the eight corners. We also summed the time the beetles stayed 0.5 cm or less from the nearest arena wall, excluding corners. Then, we compared, using a paired *t*-test, whether the arena corners or the block corners (square-root transformed) were similarly attractive to the beetles and whether they stayed more next to corners or walls. Statistics have been done using Systat v. 13 (Systat Software, Inc., San Jose, CA, USA) and Stata 15 (Stata Statistical Software, StataCorp, College Station, TX, USA).

### 2.3. Experiment 2: how do turns affect the movement of beetles?

**Figure 2.**
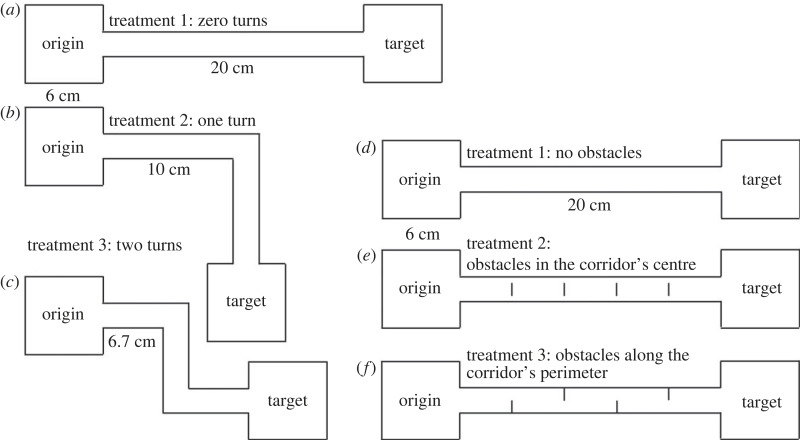
Schemes of the arenas used in (*a–c*) Experiment 2 and in (*d–f*) Experiment 3. Each arena comprised an origin cell, a target cell and a corridor. In Experiment 2, the corridor turned either once, twice or not at all (straight). In Experiment 3, the corridor comprised four obstacles in the corridor's centre, on its perimeter or no obstacles.

We implemented three treatments or arena settings, each consisting of an origin cell, a target cell (both measuring 6 × 6 cm), and a connecting corridor. The treatments differed in the corridor structure (figure 2*a–c*). Treatment 1 featured a straight corridor (20 × 2 cm) with no turns and served as the control. In treatment 2, there were two perpendicular rectangles (10 × 2 cm) that required beetles to make one turn to reach the target cell. In treatment 3, three perpendicular rectangles (6.67 × 2 cm) forced the beetles to make two turns to reach the target cell. For each beetle and treatment, we recorded whether the beetles left the origin cell (y/n). Only for those that left, we documented how many times each re-entered the origin cell and whether they reached the target cell (y/n). For the ones arriving, we documented arrival times. For those that reached the target cell, we divided the film segment to focus only on their movement within the corridor, from the moment they left the origin cell until they arrived at the target cell. The segment was divided into frames, with one frame captured every second. We used ImageJ to follow the movement of beetles in the corridor. We measured the distance moved within the corridor, the time spent in the corridor, and calculated the movement speed (distance/time). Tests were stopped either when beetles entered the origin cell or after 5 min, the earliest of the two.

First, we applied a mixed-effects logistic regression to examine whether the number of beetles leaving the origin cell differed across treatments and between sexes. We treated treatment and sex as fixed effects while using the beetle ID as a random variable. Testing for the treatment × sex interaction proved to be impossible in some cases due to a lack of variance (where all beetles left). We applied the same test to assess differences in arrival proportions at the target cell among treatments and between sexes. We compared the number of re-entries to the origin cell after leaving it (with a range of up to three times and re-entering up to four times during the test) among treatments and between sexes using a mixed-effects negative binomial regression. Focusing solely on beetles that arrived at the target cell, we employed a linear mixed model to assess differences in arrival times. We conducted three similar tests to investigate the effect of treatment and sex on the distance, time and speed within the corridor connecting the origin cell to the target cell. Owing to the deviation from a normal distribution of distance and time, they were subjected to square-root transformation.

### 2.4. Experiment 3: how do obstacles affect the movement of beetles?

We implemented three treatments, each consisting of an origin cell and a target cell (both measuring 6 × 6 cm), connected by a straight corridor (20 × 2 cm). The treatments differed in the number and location of obstacles within the corridors (figure 2*d*–*f*). Treatment 1 had no obstacles and served as the control. In treatment 2, there were four obstacles (1 cm each) uniformly distributed in the corridor's centre away from the walls. In treatment 3, the four obstacles (1 cm each) were uniformly distributed along the corridor's walls. We documented the same measurements as in Experiment 2 and similarly analysed the data.

## 3. Results

### 3.1. Experiment 1: do beetles stay longer in areas surrounded by walls?

**Figure 3.**
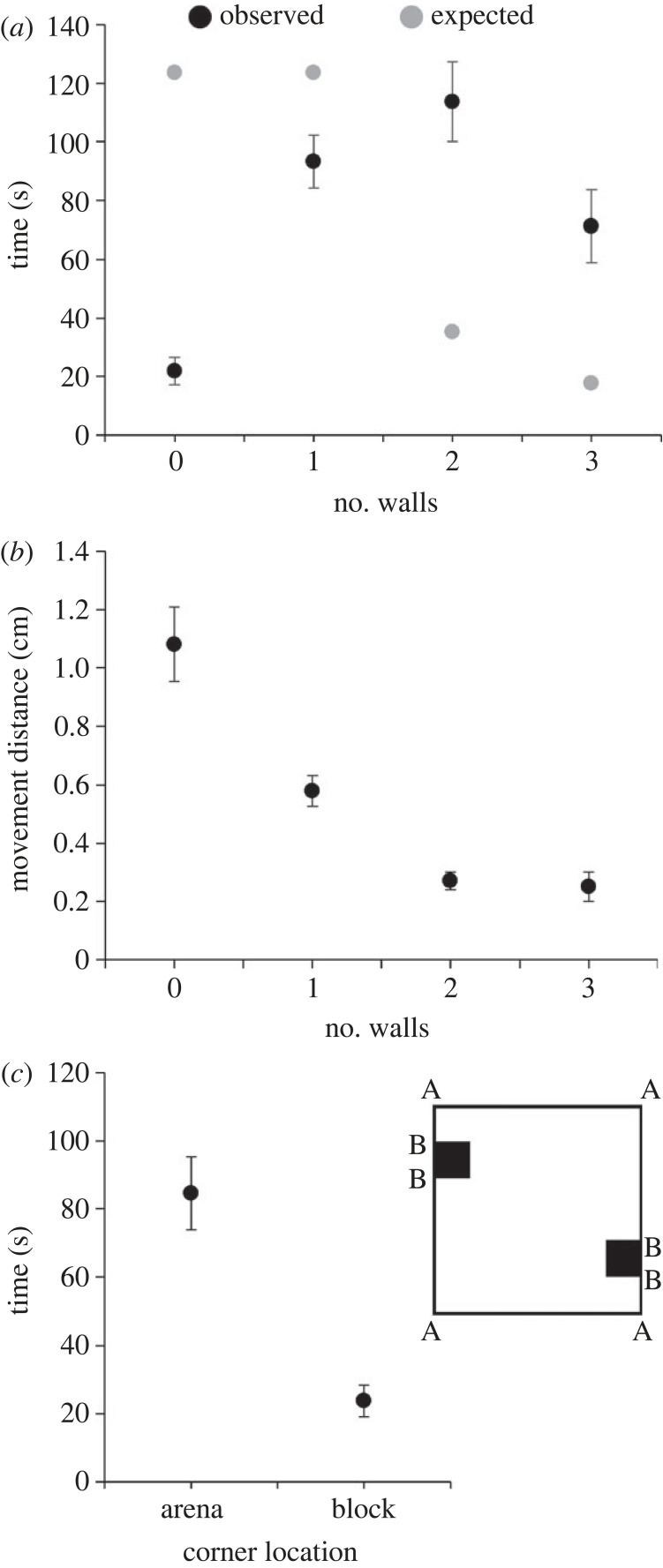
Results of Experiment 1. (*a*) The time spent (means ± 1 s.e.) in squares with 0, 1, 2 or 3 walls was not random (observed and expected times appear in black and grey, respectively). They spent the longest in squares with 2 and 3 walls (much more than expected), followed by squares with a single wall (somewhat less than expected), and the least in squares with zero walls (much less than expected). (*b*) Movement distance (means ± 1 s.e.) per second in squares with 0, 1, 2 and 3 walls. Movement distance was the shortest in squares with 2 and 3 walls and the longest in squares with zero walls. (*c*) The time spent (means ± 1 s.e.) in corners (in a radius of 0.5 cm) of the arena (marked with A) and of blocks (marked with B).

The time spent varied among square types (*F*_3,57_ = 12.772, *p* < 0.001). However, there was no effect of sex (*F*_1,18_ = 0.775, *p* = 0.390), and the two-way interaction was also not significant (*F*_3,54_ = 0.366, *p* = 0.778). The presence in square types differed more than expected (*χ*^2^ = 148.80, d.f. = 3, *p* < 0.001; figure 3*a*): Beetles stayed considerably longer than expected in squares with two and three walls (3.2 and 4.0 times longer, respectively), while they stayed shorter than expected in squares with zero and one wall (5.6 and 1.3 times less). Mean distances moved per second differed among square types (*F*_3,48_ = 33.647, *p* < 0.001; figure 3*b*): movement distances were the largest in squares with no walls, followed by squares with a single wall, and distances in squares with two or three walls were the lowest and similar between the two. There was no difference between the sexes (*F*_1,15_ = 0.293, *p* = 0.596) and the two-way interaction was not significant as well (*F*_3,45_ = 0.266, *p* = 0.849). Regarding the corners, beetles spent 108.4 ± 41.7 s (mean ± 1 s.d.) next to corners, which corresponds to 36.1 ± 13.9% of the time. This covers an area of 1.6 cm^2^ (8 × ¼ circle area, *r* = 0.5) out of the total 78 cm^2^ arena's area (9 × 9–2 × 1.5). However, arena corners were around twice as preferred as block corners (*t* = 4.166, d.f. = 19, *p* < 0.001; figure 3*c*). Beetles spent similar times next to corners and walls (corners: 36.1%, walls without corners: 41.9%; paired *t*-test: *t* = 1.122, d.f. = 19, *p* = 0.276). However, the area next to walls is almost 10 times larger than corners (approx. 15 cm^2^ versus 1.6 cm^2^).

### 3.2. Experiment 2: how do turns affect the movement of beetles?

**Figure 4.**
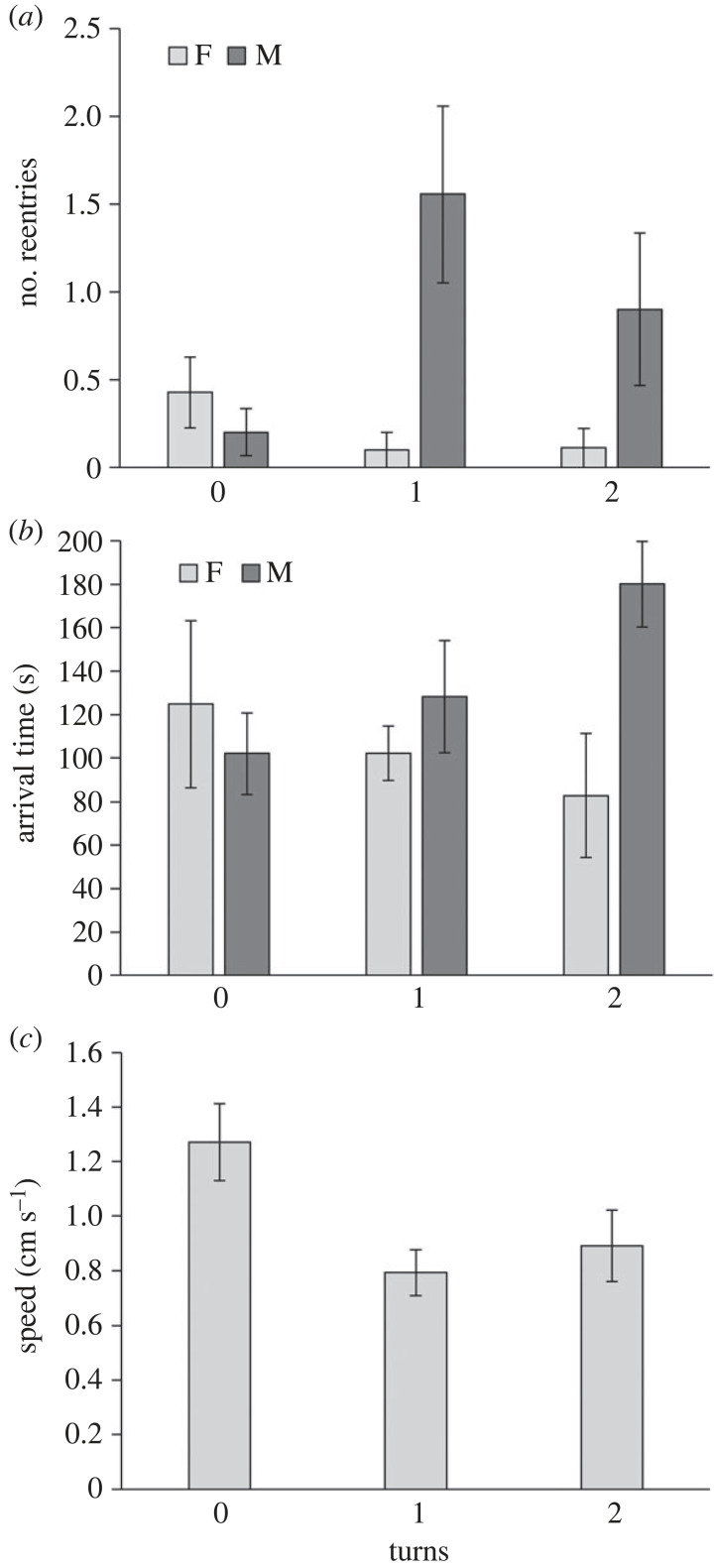
Results of Experiment 2. The effect of the number of turns of the corridor connecting the origin and the target cells on (*a*) the number of re-entries of the beetles to the origin cell after leaving it, (*b*) arrival times at the origin cell, and (*c*) movement speed in the corridor. Females and males appear in light and dark grey, respectively. Means ± 1 s.e. are presented.

The number of beetles leaving the origin cell differed neither among treatments/number of turns (*z* = 1.03, *p* = 0.304) nor between the sexes (*z* = 1.32, *p* = 0.187). Similarly, there were no differences among treatments or between the sexes in the proportion of beetles arriving at the target cell (*z* = −0.40 to −0.36, *p* = 0.689–0.717, and *z* = −0.89, *p* = 0.372 for treatment and sex, respectively). Concerning the number of re-entries to the origin cell, a significant treatment × sex interaction was observed (*z* = 2.41 and 1.95, *p* = 0.016 and 0.052; figure 4*a*): while the number of re-entries was similar between sexes in the control (no turns), in treatment 2 (one turn) and to a lesser extent in treatment 3 (two turns), males re-entered the origin cell more frequently than females. Sex and treatment were not significant as main effects (sex: *z* = −0.79, *p* = 0.432; treatment: *z* = −1.21 to −1.12, *p* = 0.226–0.262). When considering only beetles that arrived at the target cell, there was an interaction between treatment and sex that influenced arrival times (sex × treatment 3 versus 1: *t* = 2.596, *p* = 0.017; sex × treatment 2 versus 1: *t* = 1.024, *p* = 0.317; figure 4*b*): females arrived faster than males in treatment 3, while much smaller differences between the sexes were observed in treatments 1 and 2. Sex was not significant as a main effect (*t* = −0.689, *p* = 0.498) but treatment was (treatment 3 versus 1: *t* = −2.132, *p* = 0.045; treatment 2 versus 1: *t* = −0.971, *p* = 0.342). The movement speed in the corridor was higher in treatment 1 (no turns) compared with treatments 2 and 3 (one or two turns) (*t* = −2.837 *p* = 0.009 and *t* = −2.235, *p* = 0.035, respectively; figure 4*c*). Sex was not significant (*t* = −0.566, *p* = 0.577) as well as the two-way interaction (*p* > 0.492). Neither treatment, sex nor their interaction had an effect on the distance moved in the corridor (*p* > 0.126 for all). Likewise, treatment, sex and their interaction did not affect the time spent in the corridor (*p* > 0.259 for all).

### 3.3. Experiment 3: how do obstacles affect the movement of beetles?

**Figure 5.**
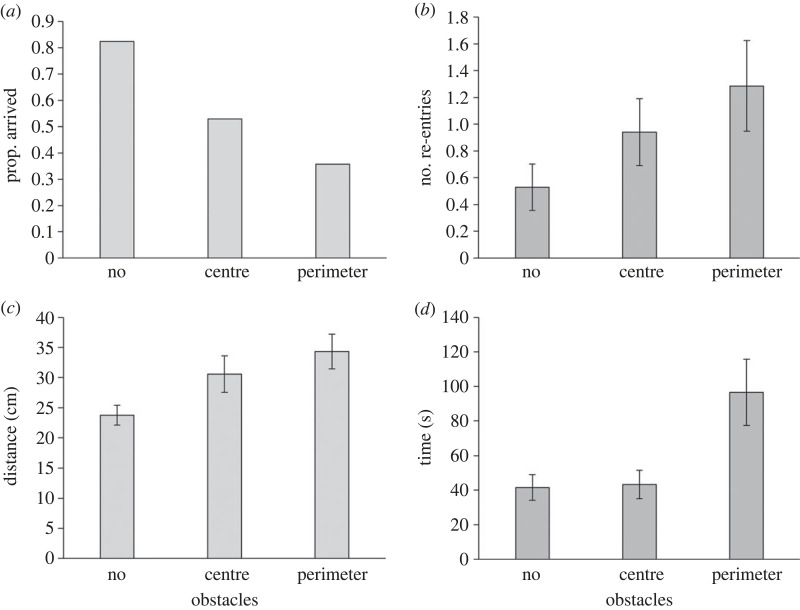
Results of Experiment 3. The effect of obstacle locations in the corridor on (*a*) the proportion of beetles arriving at the target cell, (*b*) the number of re-entries of the beetles to the origin cell after leaving it, and (*c,d*) the distance and time moved in the corridor. Means ± 1 s.e. are presented.

The number of beetles leaving the origin cell differed neither among treatments/the number of obstacles and their location (*z* = −1.14, *p* = 0.256 and *z* = 0.00, *p* = 1.00 for treatment 1 versus 3 and 1 versus 2) nor between the sexes (*z* = −1.29, *p* = 0.197). More beetles arrived at the target cell in treatment 1 (no obstacles) than in treatment 3 (obstacles along the corridor's perimeter), with treatment 2 (obstacles in the corridor's centre) in between (treatment 1 versus 3: *z* = −2.28, *p* = 0.023; treatment 1 versus 2: −1.66, *p* = 0.096; figure 5*a*). There was neither an effect of sex nor an effect of the interaction (sex: *z* = −1.00, *p* = 0.317; interaction: *p* > 0.324). Regarding the number of re-entries to the origin cell, there was a difference among treatments with the fewest re-entries in treatment 1 and the most frequent re-entries in treatment 3 (treatment 1 versus 3: *z* = 2.13, *p* = 0.033; treatment 1 versus 2: *z* = 1.42, *p* = 0.156; figure 5*b*). There was no difference between the sexes (*z* = −0.86, *p* = 0.391). Regarding only beetles that arrived at the target cell, there was neither a difference among treatments (treatment 1 versus 3: *t* = 0.032, *p* = 0.976; treatment 1 versus 2: *z* = 0.630, *p* = 0.544) nor an effect of sex (*t* = 0.528, *p* = 0.611) nor an effect of the interaction (*p* > 0.069). Movement distances in the corridor were the highest in treatment 3 followed by treatment 2 and the lowest in treatment 1 (treatment 1 versus 3: *t* = 3.107, *p* = 0.009; treatment 1 versus 2: *t* = 2.366, *p* = 0.036; figure 5*c*). Sex and the two-way interaction were not significant (sex: *t* = 0.439, *p* = 0.669; interaction: *p* > 0.426). Time in the corridor was longer in treatment 3 than in treatment 1 (*t* = 3.141, *p* = 0.009; figure 5*d*), with no difference between treatment 2 and 1 (*t* = 0.250, *p* = 0.807). Neither sex nor the two-way interaction was significant (sex: *t* = −0.345, *p* = 0.736; interaction: *p* > 0.595). Finally, movement speed was not affected by treatment (treatment 1 versus 3: *t* = −1.770, *p* = 0.102; treatment 1 versus 2: *t* = 0.451, *p* = 0.660), sex (*t* = 0.678, *p* = 0.510) or their interaction (*p* > 0.444).

## 4. Discussion

We present here evidence of flour beetles' preference for corners over walls, supported by the proportion of time near corners relative to all other parts of the arena and the lower movement speed in or near corners. The presence of an additional nearby corner (i.e. squares of three walls rather than only two) did not influence the preference for specific corners. Beetles seem to avoid open areas as much as possible and use walls to direct themselves to corners, where they tend to stay much longer. Movement between an origin cell and a target cell was impacted by the corridor setting, influencing overall movement and the probability of reaching the target. The movement speed was lower in corridors with one or two turns compared with a straight corridor, and the number of re-entries to the origin cell after departure was higher. Obstacles along the corridor prevented or delayed arrival at the target cell and increased the distance travelled in the corridor. Obstacles on the corridor's perimeter were more influential than those at the corridor's centre probably because the beetles tended to follow walls.

In the first experiment, the test arena was divided into 34 squares (plus two non-accessible ones) measuring 1.5 × 1.5 cm each, with varying numbers of walls surrounding them, ranging from zero to three. The avoidance of squares with no walls is clear: the beetles stayed there the least and moved the fastest through these squares. Despite previous studies considering red flour beetles as highly thigmotactic, beetles were less frequently present in squares with a single wall than expected. Movement speed in such squares was lower than in squares with no walls but higher than that in squares with two or three walls. The conclusion is that the red flour beetle prefers more strongly corners than it is thigmotactic or favours walls. Other studies reached the same conclusion in the same flour beetle and other tested arthropods but the exact preference depends also on the availability of cover, food and illumination [33,49].

There was no difference in terms of beetle presence and movement speed between squares with two or three walls. This suggests that the preference is indeed for corners, regardless of the proximity of third wall. It is possible that the distance to the third wall (1.5 cm) is too great, and different results might have been observed with a shorter distance. Many small animals prefer to hide in crevices and can use walls to find and reach them [58–60]. Thus, it could be that the three-wall cells were not tight enough to be perceived as a shelter. Comparing the four arena corners and the four block corners (figure 3*c*), beetles stayed longer near the former ones. The reason is probably the longer two walls that lead to the arena corners than the shorter two walls leading to the block corners. The longer the wall is the higher chance there is for beetles to bump into it. When they do so, they follow it until they reach a corner, where they stay for a while. Thus, by chance, longer walls ‘drain' more beetles to corners than shorter ones.

In the second and third experiments, we examined how the characteristics of the corridor, connecting the origin and target cells, affect the arrival patterns of beetles. In the third experiment, the corridor was obstructed by obstacles either in its centre or along its perimeter. The effects were evident: obstacles decreased the likelihood of beetles reaching the target cell, increased the chances of beetles returning to the origin cell, and extended both the distance and time spent in the corridor. This fits research on other insects showing an increase in travel time or a decrease in speed with habitat complexity or an increasing number of obstacles in the habitat [22–24,61]. It also fits a previous study on flour beetles showing that corridor length and steepness negatively affect dispersal success [62]. The obstacles in the corridors and their turn forced animals to turn too as they would have otherwise bumped into walls. Frequent turning forces animals to slow down and increases the energetic cost of movement [25,63]. Obstacles along the corridor's perimeter had a more pronounced impact on all measurements (figure 5). This is perhaps because the beetles prefer to move along the corridor's perimeter, which is prevented to some extent by obstacles on it.

Inter-sexual differences in movement were uncommon in our experiments. We observed such differences in the second experiment, which involved turns. Here, the arrival times of males were longer than those of females when the corridors featured turns, but slightly longer for females in a straight corridor. In other words, males dealt less well with turns. This contradicts to some extent the common assumption that males are better in solving spatial tasks than females and that males are more sensitive to the microhabitat's shape than females [64,65]. Past studies on the red flour beetle disagreed on whether individuals of one sex move more than the other (females move more than males [51,66]; males move more than females [53,67]; no difference [35,68]). Whereas males may move over longer distances in search of females, females may do so in search of suitable oviposition sites [49]. Since movement is influenced by various factors, including leg length, age, mating status, physiological status, population and circadian rhythm [48,51,54,55,69,70], it is likely that inter-sexual differences are context-specific. Finally, we might have missed several more subtle differences between the sexes or treatments owing to our relatively low sample (10 per sex).

Our study might have practical implications regarding how to address the spread of insect pests. For example, Experiment 1 suggests the optimal trap location—corners that intersect long walls. Other studies raised similar suggestions of placing traps for pests along long walls or in corners [71–74]. If corners or obstacles slow the beetles down, delay them a bit or force the beetles to move along them, then traps placed there may be more attractive. Additionally, this study implies that obstacles positioned along walls may disturb the dispersal of flour beetles to a greater extent than those located in the habitat's centre. The practical implication of obstacles depends on the number of dispersing pests. If the number of dispersers is small, then obstacles might be useful in preventing dispersal. Even a single individual (a mated female) can have great negative impact on a new food patch (e.g. [75]). An additional methodological implication strengthens the conclusion of our previous study demonstrating strong effects of the test arena's shape on a variety of movement characteristics [35]. Whereas in round arenas movement along the perimeter is frequent (greater than 80% of the time), there is a strong preference in square arenas, like the one used here, for corners, and movement along the perimeter (or walls) is less frequent than expected by chance. Thus, the arena shape modifies movement and the preference for specific arena sections not only quantitatively but qualitatively.

## Data Availability

The data are provided in the electronic supplementary material [76].

## References

[1] Mitchell WA, Lima SL. 2002 Predator-prey shell games: large-scale movement and its implications for decision-making by prey. Oikos **99**, 249-259. (10.1034/j.1600-0706.2002.990205.x)

[2] Nathan R, Getz WM, Revilla E, Holyoak M, Kadmon R, Saltz D, Smouse PE. 2008 A movement ecology paradigm for unifying organismal movement research. Proc. Natl Acad. Sci. USA **105**, 19 052-19 059. (10.1073/pnas.0800375105) PMC2614714 19060196

[3] Hawkes C. 2009 Linking movement behaviour, dispersal and population processes: is individual variation a key? J. Anim. Ecol. **78**, 894-906. (10.1111/j.1365-2656.2009.01534.x) 19302396

[4] Shepard EL, Wilson RP, Rees WG, Grundy E, Lambertucci SA, Vosper SB. 2013 Energy landscapes shape animal movement ecology. Am. Nat. **182**, 298-312. (10.1086/671257) 23933722

[5] Goossens S, Wybouw N, Van Leeuwen T, Bonte D. 2020 The physiology of movement. Mov. Ecol. **8**, 5. (10.1186/s40462-020-0192-2) 32042434 PMC7001223

[6] Williams HJ, Safi K. 2021 Certainty and integration of options in animal movement. Trends Ecol. Evol. **36**, 990-999. (10.1016/j.tree.2021.06.013) 34303526

[7] Eggleston DB, Lipcius RN. 1992 Shelter selection by spiny lobster under variable predation risk, social conditions, and shelter size. Ecology **73**, 992-1011. (10.2307/1940175)

[8] Fero KC, Moore PA. 2014 Shelter availability influences social behavior and habitat choice in crayfish, *Orconectes virilis*. Behaviour **151**, 103-123. (10.1163/1568539X-00003125)

[9] Götz KG, Biesinger R. 1985 Centrophobism in *Drosophila melanogaster*. I. Behavioral modification induced by ether. J. Comp. Physiol. A **156**, 319-327. (10.1007/BF00610725)

[10] Dussutour A, Deneubourg JL, Fourcassié V. 2005 Amplification of individual preferences in a social context: the case of wall-following in ants. Proc. R. Soc. B **272**, 705-714. (10.1098/rspb.2004.2990) PMC1602045 15870033

[11] Kallai J, Makany T, Csatho A, Karadi K, Horvath D, Kovacs-Labadi B, Jarai R, Lynn N, Jacobs JW. 2007 Cognitive and affective aspects of thigmotaxis strategy in humans. Behav. Neurosci. **121**, 21-30. (10.1037/0735-7044.121.1.21) 17324048

[12] Harris AP, D'Eath RB, Healy SD. 2009 Environmental enrichment enhances spatial cognition in rats by reducing thigmotaxis (wall hugging) during testing. Anim. Behav. **77**, 1459-1464. (10.1016/j.anbehav.2009.02.019)

[13] McMahon A, Patullo BW, Macmillan DL. 2005 Exploration in a T-maze by the crayfish *Cherax destructor* suggests bilateral comparison of antennal tactile information. Biol. Bull. **208**, 183-188. (10.2307/3593150) 15965123

[14] Lamprea MR, Cardenas FP, Setem J, Morato S. 2008 Thigmotactic responses in an open-field. Braz. J. Med. Biol. Res. **41**, 135-140. (10.1590/S0100-879X2008000200010) 18297193

[15] Borba JV, Biasuz E, Sabadin GR, Savicki AC, Canzian J, Luchiari AC, Adedara IA, Rosemberg DB. 2022 Influence of acute and unpredictable chronic stress on spatio-temporal dynamics of exploratory activity in zebrafish with emphasis on homebase-related behaviors. Behav. Brain Res. **435**, 114034. (10.1016/j.bbr.2022.114034) 35914633

[16] Scharf I, Farji-Brener A. In press. Wall-following behavior: its ultimate and proximate explanations, prevalence, and implications. Adv. Study Behav.

[17] Alzogaray RA, Fontán A, Zerba EN. 1997 Evaluation of hyperactivity produced by pyrethroid treatment on third instar nymphs of *Triatoma infestans* (Hemiptera: Reduviidae). Arch. Insect Biochem. Physiol. **35**, 323-333. (10.1002/(SICI)1520-6327(199705)35:3<323::AID-ARCH6>3.0.CO;2-U) 9177136

[18] Camhi JM, Johnson EN. 1999 High-frequency steering maneuvers mediated by tactile cues: antennal wall-following in the cockroach. J. Exp. Biol. **202**, 631-643. (10.1242/jeb.202.5.631) 9929464

[19] Douglass NJ, Reinert HK. 1982 The utilization of fallen logs as runways by small mammals. Proc. Natl Acad. Sci. USA **56**, 162-164.

[20] Jethva S, Liversage K, Kundu R. 2022 Does topography of rocky intertidal habitat affect aggregation of cerithiid gastropods and co-occurring macroinvertebrates? Oceanologia **64**, 387-395. (10.1016/j.oceano.2022.01.006)

[21] Taniguchi H, Tokeshi M. 2004 Effects of habitat complexity on benthic assemblages in a variable environment. Freshw. Biol. **49**, 1164-1178. (10.1111/j.1365-2427.2004.01257.x)

[22] Torres-Contreras H, Vasquez RA. 2007 Spatial heterogeneity and nestmate encounters affect locomotion and foraging success in the ant *Dorymyrmex goetschi*. Ethology **113**, 76-86. (10.1111/j.1439-0310.2006.01302.x)

[23] Radnan GN, Gibb H, Eldridge DJ. 2018 Soil surface complexity has a larger effect on food exploitation by ants than a change from grassland to shrubland. Ecol. Entomol. **43**, 379-388. (10.1111/een.12510)

[24] Bega D, Samocha Y, Yitzhak N, Saar M, Subach A, Scharf I. 2019 The effect of maze complexity on maze-solving time in a desert ant. Behav. Proc. **166**, 103893. (10.1016/j.beproc.2019.103893) 31252072

[25] Wilson RP, Griffiths IW, Legg PA, Friswell MI, Bidder OR, Halsey LG, Lambertucci SA, Shepard ELC. 2013 Turn costs change the value of animal search paths. Ecol. Lett. **16**, 1145-1150. (10.1111/ele.12149) 23848530

[26] Wilson RP et al. 2021 Path tortuosity changes the transport cost paradigm in terrestrial animals. Ecography **44**, 1524-1532. (10.1111/ecog.05850)

[27] Petren K, Case TJ. 1998 Habitat structure determines competition intensity and invasion success in gecko lizards. Proc. Natl Acad. Sci. USA **95**, 11 739-11 744. (10.1073/pnas.95.20.11739) PMC21710 9751735

[28] Finke DL, Denno RF. 2002 Intraguild predation diminished in complex-structured vegetation: implications for prey suppression. Ecology **83**, 643-652. (10.1890/0012-9658(2002)083[0643:IPDICS]2.0.CO;2)

[29] Gratwicke B, Speight MR. 2005 The relationship between fish species richness, abundance and habitat complexity in a range of shallow tropical marine habitats. J. Fish Biol. **66**, 650-667. (10.1111/j.0022-1112.2005.00629.x)

[30] Martinez R, Morato S. 2004 Thigmotaxis and exploration in adult and pup rats. Rev. Etol. **6**, 49-54.

[31] Zadicario P, Avni R, Zadicario E, Eilam D. 2005 ‘Looping’ – an exploration mechanism in a dark open field. Behav. Brain Res. **159**, 27-36. (10.1016/j.bbr.2004.09.022) 15794994

[32] Soibam B, Mann M, Liu L, Tran J, Lobaina M, Kang YY, Gunaratne GH, Pletcher S, Roman G. 2012 Open-field arena boundary is a primary object of exploration for *Drosophila*. Brain Behav. **2**, 97-108. (10.1002/brb3.36) 22574279 PMC3345355

[33] Tapia F, Olivares J, Schmachtenberg O. 2020 The visual spectral sensitivity of the Chilean recluse spider *Loxosceles laeta*. J. Exp. Biol. **223**, jeb217133. 31852757 10.1242/jeb.217133

[34] Mon-Williams M, Tresilian JR, Coppard VL, Carson RG. 2001 The effect of obstacle position on reach-to-grasp movements. Exp. Brain Res. **137**, 497-501.. (10.1007/s002210100684) 11355394

[35] Scharf I, Hanna K, Gottlieb D. In press. Experimental arena settings might lead to misinterpretation of movement properties. Insect Sci. (10.1111/1744-7917.13213) 37231528

[36] Swingland IR. 1983 Intraspecific differences in movement. In The ecology of animal movement (eds IR Swingland, PJ Greenwood), pp. 102-115. Oxford, UK: Oxford University Press.

[37] Jones CM, Braithwaite VA, Healy SD. 2003 The evolution of sex differences in spatial ability. Behav. Neurosci. **117**, 403-411. (10.1037/0735-7044.117.3.403) 12802870

[38] Martin JR. 2004 A portrait of locomotor behaviour in *Drosophila* determined by a video-tracking paradigm. Behav. Proc. **67**, 207-219. (10.1016/j.beproc.2004.04.003) 15240058

[39] Brand JA, Henry J, Melo GC, Wlodkowic D, Wong BB, Martin JM. 2023 Sex differences in the predictability of risk-taking behavior. Behav. Ecol. **34**, 108-116. (10.1093/beheco/arac105) 36789395 PMC9918862

[40] Perrot-Sinal TS, Kostenuik MA, Ossenkopp KP, Kavaliers M. 1996 Sex differences in performance in the Morris water maze and the effects of initial nonstationary hidden platform training. Behav. Neurosci. **110**, 1309-1320. (10.1037/0735-7044.110.6.1309) 8986334

[41] Ansai S, Hosokawa H, Maegawa S, Kinoshita M. 2016 Chronic fluoxetine treatment induces anxiolytic responses and altered social behaviors in medaka, *Oryzias latipes*. Behav. Brain Res. **303**, 126-136. (10.1016/j.bbr.2016.01.050) 26821288

[42] Atta B, Rizwan M, Sabir AM, Gogi MD, Ali K. 2020 Damage potential of *Tribolium castaneum* (Herbst) (Coleoptera: Tenebrionidae) on wheat grains stored in hermetic and non-hermetic storage bags. Int. J. Trop. Insect Sci. **40**, 27-37. (10.1007/s42690-019-00047-0)

[43] Vidan E, Quinn E, Trostanetsky A, Rapaport A, Doron J, Harush A, Kostyukovsky M, Gottlieb D. 2020 How do similar community dynamics yield different population dynamics and spatial distributions of species? J. Stored Prod. Res. **87**, 101621. (10.1016/j.jspr.2020.101621)

[44] Brown SJ, Shippy TD, Miller S, Bolognesi R, Beeman RW, Lorenzen MD, Bucher G, Wimmer EA, Klingler M. 2009 The red flour beetle, *Tribolium castaneum* (Coleoptera): a model for studies of development and pest biology. Cold Spring Harb. Protoc. **2009**, pdb-emo126. (10.1101/pdb.emo126) 20147228

[45] Campbell JF, Athanassiou CG, Hagstrum DW, Zhu KY. 2022 *Tribolium castaneum*: a model insect for fundamental and applied research. Annu. Rev. Entomol. **67**, 347-365. (10.1146/annurev-ento-080921-075157) 34614365

[46] Ridley AW, Hereward JP, Daglish GJ, Raghu S, Collins PJ, Walter GH. 2011 The spatiotemporal dynamics of *Tribolium castaneum* (Herbst): adult flight and gene flow. Mol. Ecol. **20**, 1635-1646. (10.1111/j.1365-294X.2011.05049.x) 21375637

[47] Semeao AA, Campbell JF, Whitworth RJ, Sloderbeck PE. 2013 Movement of *Tribolium castaneum* within a flour mill. J. Stored Prod. Res. **54**, 17-22. (10.1016/j.jspr.2013.03.004)

[48] Arnold PA, Cassey P, White CR. 2017 Functional traits in red flour beetles: the dispersal phenotype is associated with leg length but not body size nor metabolic rate. Funct. Ecol. **31**, 653-661. (10.1111/1365-2435.12772)

[49] Campbell JF, Hagstrum DW. 2002 Patch exploitation by *Tribolium castaneum*: movement patterns, distribution, and oviposition. J. Stored Prod. Res. **38**, 55-68. (10.1016/S0022-474X(00)00042-4)

[50] Campbell JF. 2013 Influence of landscape pattern in flour residue amount and distribution on *Tribolium castaneum* (Herbst) response to traps baited with pheromone and kairomone. J. Stored Prod. Res. **52**, 112-117. (10.1016/j.jspr.2012.11.004)

[51] Wexler Y, Subach A, Pruitt JN, Scharf I. 2016 Behavioral repeatability of flour beetles before and after metamorphosis and throughout aging. Behav. Ecol. Sociobiol. **70**, 745-753. (10.1007/s00265-016-2098-y)

[52] Wexler Y, Scharf I. 2017 Distinct effects of two separately applied stressors on behavior in the red flour beetle. Behav. Proc. **145**, 86-92. (10.1016/j.beproc.2017.10.008) 29107019

[53] Arnold PA, Cassey P, White CR. 2023 Morphological shifts in response to spatial sorting on dispersal behaviour in red flour beetles across multiple generations. J. Zool **320**, 131-142. (10.1111/jzo.13062)

[54] Arnold PA, Cassey P, White CR. 2016 Maturity matters for movement and metabolic rate: trait dynamics across the early adult life of red flour beetles. Anim. Behav. **111**, 181-188. (10.1016/j.anbehav.2015.10.023)

[55] Gilad T, Koren R, Moalem Y, Subach A, Scharf I. 2018 Effect of continuous and alternating episodes of starvation on behavior and reproduction in the red flour beetle. J. Zool. **305**, 213-222. (10.1111/jzo.12556)

[56] Halliday WD, Blouin-Demers G. 2014 Red flour beetles balance thermoregulation and food acquisition via density-dependent habitat selection. J. Zool. **294**, 198-205. (10.1111/jzo.12168)

[57] Abràmoff MD, Magalhães PJ, Ram SJ. 2004 Image processing with ImageJ. Biophotonics Int. **11**, 36-42.

[58] Burrows MT, Kawai K, Hughes RN. 1999 Foraging by mobile predators on a rocky shore: underwater TV observations of movements of blennies *Lipophrys pholis* and crabs *Carcinus maenas*. Mar. Ecol. Prog. Ser. **187**, 237-250. (10.3354/meps187237)

[59] Lorenzo MG, Lazzari CR. 1999 Temperature and relative humidity affect the selection of shelters by *Triatoma infestans*, vector of Chagas disease. Acta Trop. **72**, 241-249. (10.1016/S0001-706X(98)00094-1) 10232780

[60] Masarovič R, Zvaríková M, Fedorová J, Fedor P. 2017 Thigmotactic behavior of *Limothrips cerealium* (Thysanoptera: Thripidae) leads to laboratory equipment damage in the Czech Republic. J. Entomol. Sci. **52**, 308-310.

[61] Gibb H, Parr CL. 2010 How does habitat complexity affect ant foraging success? A test using functional measures on three continents. Oecologia **164**, 1061-1073. (10.1007/s00442-010-1703-4) 20589395

[62] Arnold PA, Rafter MA, Malekpour R, Cassey P, Walter GH, White CR. 2017 Investigating movement in the laboratory: dispersal apparatus designs and the red flour beetle, *Tribolium castaneum*. Entomol. Exp. Appl. **163**, 93-100. (10.1111/eea.12551)

[63] Wynn ML, Clemente C, Nasir A, Wilson RS. 2015 Running faster causes disaster: trade-offs between speed, manoeuvrability and motor control when running around corners in northern quolls (*Dasyurus hallucatus*). J. Exp. Biol. **218**, 433-439. (10.1242/jeb.111682) 25653423

[64] Rodríguez CA, Chamizo VD, Mackintosh NJ. 2011 Overshadowing and blocking between spatial orientation landmark learning and shape learning: the importance of sex differences. Learn. Behav. **39**, 324-335. (10.3758/s13420-011-0027-5) 21472414

[65] Chamizo VD, Rodrigo T. 2022 Spatial orientation. In Encyclopedia of animal cognition and behavior (eds J Vonk, TK Shackelford), pp. 6592-6602. Cham, Switzerland: Springer International Publishing.

[66] Matsumura K, Miyatake T. 2015 Differences in attack avoidance and mating success between strains artificially selected for dispersal distance in *Tribolium castaneum*. PLoS ONE **10**, e0127042. (10.1371/journal.pone.0127042) 25970585 PMC4430303

[67] Ritte U, Lavie B. 1977 The genetic basis of dispersal behavior in the flour beetle *Tribolium castaneum*. Can. J. Genet. Cytol. **19**, 717-722. (10.1139/g77-078)

[68] Cronin JT, Goddard J, Krivchenia A, Shivaji R. 2023 Density-dependent within-patch movement behavior of two competing species. Ecol. Evol. **13**, e10753. (10.1002/ece3.10753) 38020706 PMC10659955

[69] Wexler Y, Wertheimer KO, Subach A, Pruitt JN, Scharf I. 2017 Mating alters the link between movement activity and pattern in the red flour beetle. Physiol. Entomol. **42**, 299-306. (10.1111/phen.12195)

[70] Abe MS, Matsumura K, Yoshii T, Miyatake T. 2021 Amplitude of circadian rhythms becomes weaken in the north, but there is no cline in the period of rhythm in a beetle. PLoS ONE **16**, e0245115. 33444354 10.1371/journal.pone.0245115 PMC7808652

[71] Durier V, Rivault C. 2003 Exploitation of home range and spatial distribution of resources in German cockroaches (Dictyoptera: Blattellidae). J. Econ. Entomol. **96**, 1832-1837. (10.1093/jee/96.6.1832) 14977123

[72] Toews MD, Arthur FH, Campbell JF. 2005 Role of food and structural complexity on capture of *Tribolium castaneum* (Herbst) (Coleoptera: Tenebrionidae) in simulated warehouses. Environ. Entomol. **34**, 164-169. (10.1603/0046-225X-34.1.164)

[73] Buckman KA, Campbell JF. 2013 How varying pest and trap densities affect *Tribolium castaneum* capture in pheromone traps. Entomol. Exp. Appl. **146**, 404-412. (10.1111/eea.12039)

[74] Kells SA, Hymel SN. 2017 The influence of time and distance traveled by bed bugs, *Cimex lectularius*, on permethrin uptake from treated mattress liners. Pest Manag. Sci. **73**, 113-117. (10.1002/ps.4294) 27098708

[75] Campbell JF, Runnion C. 2003 Patch exploitation by female red flour beetles, *Tribolium castaneum*. J. Insect Sci. **3**, 20. 15841236 10.1093/jis/3.1.20 PMC524659

[76] Scharf I, Radai A, Goldshtein D, Hanna K. 2024 Flour beetles prefer corners over walls and are slowed down with increasing habitat complexity. Figshare. (10.6084/m9.figshare.c.7007830) PMC10791520 38234433

